# Cardiac Glycosides as Immune System Modulators

**DOI:** 10.3390/biom11050659

**Published:** 2021-04-29

**Authors:** Jan Škubník, Vladimíra Pavlíčková, Silvie Rimpelová

**Affiliations:** Department of Biochemistry and Microbiology, University of Chemistry and Technology Prague, Technická 3, 166 28 Prague 6, Czech Republic; jan.skubnik@vscht.cz (J.Š.); vladimira.pavlickova@vscht.cz (V.P.)

**Keywords:** cardiac steroids, sodium-potassium ATPase, NKA signalosome, immunogenic cell death, inflammation, retinoic acid receptor-related orphan receptor γ thymus, interleukin 17, Th17, calreticulin, anticancer compounds

## Abstract

Cardiac glycosides (CGs) are natural steroid compounds occurring both in plants and animals. They are known for long as cardiotonic agents commonly used for various cardiac diseases due to inhibition of Na^+^/K^+^-ATPase (NKA) pumping activity and modulating heart muscle contractility. However, recent studies show that the portfolio of diseases potentially treatable with CGs is much broader. Currently, CGs are mostly studied as anticancer agents. Their antiproliferative properties are based on the induction of multiple signaling pathways in an NKA signalosome complex. In addition, they are strongly connected to immunogenic cell death, a complex mechanism of induction of anticancer immune response. Moreover, CGs exert various immunomodulatory effects, the foremost of which are connected with suppressing the activity of T-helper cells or modulating transcription of many immune response genes by inhibiting nuclear factor kappa B. The resulting modulations of cytokine and chemokine levels and changes in immune cell ratios could be potentially useful in treating sundry autoimmune and inflammatory diseases. This review aims to summarize current knowledge in the field of immunomodulatory properties of CGs and emphasize the large area of potential clinical use of these compounds.

## 1. Introduction

According to the Global Cancer Observatory study (GLOBOCAN) 2020, it is estimated that in 2020, there was an increase of 19.3 million new cases of cancer, half of which lead to patient death. Based on the prediction models, in 2040, the global cancer burden is expected to rise to 28.4 million cases, which would mean a 46% increase in comparison to 2020 [1]. These very negative expectations put extreme pressure on the development of novel anticancer drugs. However, the evaluation process of completely new substances ranging from compound synthesis, through preclinical and clinical trials, to everyday clinical use, is very lengthy. A much more time- and cost-efficient approach to obtain a “novel” anticancer drug is to screen substances, which have been already approved for other indications than cancer, and, thus, they have been proven safe. Such an approach is called drug-repositioning, or drug-repurposing. Among such potential repurposed drug candidates belong, for example, cardiac glycosides (CGs), currently used in clinical practice for the treatment of heart diseases, such as congestive heart failure or cardiac arrhythmias. Furthermore, CGs also exert very promising anticancer activity both in vitro and in vivo [2,3,4,5,6,7,8,9,10,11,12,13,14,15,16,17,18], which is based mainly on the induction of complex signaling cascades in Na^+^/K^+^-ATPase (NKA) signalosome and induction of immune response. This fact could be utilized not only for cancer treatment but also in the treatment of a plethora of autoimmune and inflammatory diseases. Based on the immunomodulatory properties of CGs, they represent a promising multifunctional treatment modality.

## 2. Cellular Targets of Cardiac Glycosides

CGs are natural steroid compounds, which occur mainly in plants, such as foxgloves (*Digitalis* sp.) [19,20,21,22] or oleanders (*Nerium* sp.) [23,24,25,26], but also animals, e.g., in the skin of particular amphibians such as toads (*Bufa bufa* and others) [27,28]. Besides, there are equivalents of CGs also in humans, referred to as digitalis-like factors [29,30]. CGs from all plants, animals, and humans have a conserved structure, which is composed of a steroid core, a lactone ring, and a sugar moiety. Depending on the lactone ring, two major groups of CGs are distinguished: cardenolides, which have a 5-membered lactone ring, and bufadienolides with a 6-membered lactone ring. Cardenolides, for example, digitoxin, digoxin, or oleandrin, are found mainly in plants, whereas bufadienolides, such as bufalin or cinobufagin, occur mainly in animals. The variations of the particular compounds are mostly ensured by different compositions of sugars. CGs include commonly occurring sugars, such as glucose, galactose, mannose, or rhamnose, but also very specific ones such as digitoxose, thevetose, cymarose, allose, or altrose [31]. The constitution of the substances naturally impacts their biological properties, mainly the duration of the inhibition of NKA, which is the key cellular target of CGs.

**Figure 1:**
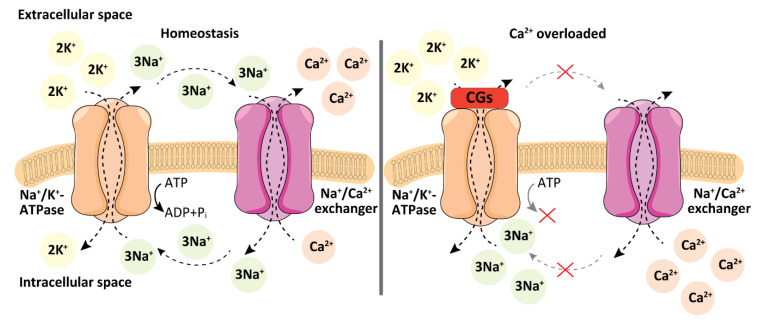
Physiological action of Na^+^/K^+^-ATPase and Na^+^/Ca^2+^ exchanger compared to the action after treatment with cardiac glycosides (CGs). In homeostasis, Na^+^/K^+^-ATPase pumps three Na^+^ ions out of a cell and two K^+^ ions into the cell. Physiologically Na^+^/Ca^2+^ exchanger imports three Na^+^ ions back into the cell in exchange for one Ca^2+^ ion. When the activity of Na^+^/K^+^ ATPase is blocked by CGs, Na^+^ ions accumulate inside the cell, which leads to a subsequent increase in intracellular Ca^2+^ ion concentration due to activation of the Na^+^/Ca^2+^ exchanger. Inspired and adapted according to ref. [34].

NKA is a transmembrane protein with enzyme activity. It usually forms a dimer, which is composed of different isoforms of α and β subunits depending on specific tissue. NKA acts as an antiport transporter of Na^+^ and K^+^ ions through the plasma membrane. This transport is ensured by energy from adenosine triphosphate (ATP) cleavage and it is crucial for the maintenance of the cell plasma membrane potential. The CG binding to NKA impedes this function, which leads to the accumulation of Na^+^ ions in the cytosol [32]. As a consequence, the Na^+^/Ca^2+^ exchanger is activated and the intracellular concentration of Ca^2+^ increases (Figure 1). The Ca^2+^ ions play an important role in heart muscle contractility. They bind and conformationally change troponin, which in the relaxed state of the muscle prevents actin-myosin contraction. Thereby, they ensure the unmasking of myosin for its binding to actin. [33]. Thus, CGs directly affect heart muscle contractility, which is the main reason for their use in cardiology.

**Figure 2:**
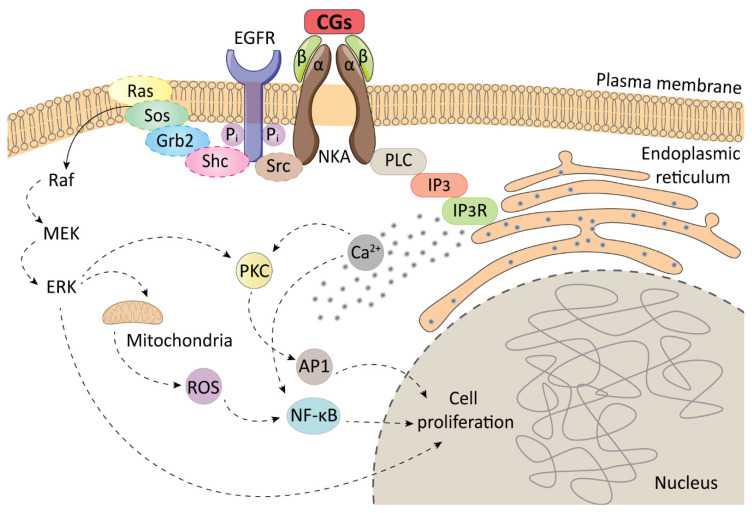
A schematic representation of the Na^+^/K^+^-ATPase (NKA) signalosome. Adapted from [45]. AP1–transcription factor; CGs–cardiac glycosides; EGFR-epidermal growth factor receptor; ERK- extracellular signal-regulated kinase; Grb2-growth factor receptor-bound protein 2; IP_3_–inositol triphosphate; IP3R–inositol triphosphate receptor; MEK-mitogen-activated protein kinase kinase; NF-κB-nuclear factor kappa-light-chain-enhancer of activated B cells; PKC–protein kinase C; PLC–phospholipase C; Raf–serine/threonine kinase; Ras–rat sarcoma protein; ROS–reactive oxygen species; Shc–Src homology 2 domain-containing transforming protein 1; Sos–son of sevenless; Src-non-receptor tyrosine kinase.

The aforementioned inhibition of NKA pumping activity occurs at high therapeutic concentrations of CGs (hundreds of nM) [32]. However, when lower CG concentrations of the compounds are used, a complex signalosome is formed with NKA in its center. Upon the lower CG concentrations, NKA, besides pumping, plays an important role also as a receptor. The very complex cascade of NKA signalosome (Figure 2), has been recently extensively reviewed elsewhere, see refs. [35,36,37]. In brief, after stimulation of NKA by CGs, protein tyrosine phosphorylation has been detected, though, NKA does not include a kinase domain. It is now well-established that this kinase activity is ensured by membrane-associated non-receptor tyrosine kinases from the Src family, which are regulated by the α1-subunit of NKA. The binding of CGs to NKA, thus, indirectly activates further transduction cascade, since NKA/Src is linked to several other partners, the most important of which are the epidermal growth factor receptors (EGFR). Activation of EGFR by phosphorylation leads to recruitment of adaptor Src-homology domain-containing protein, which then binds to a protein complex of growth factor receptor-bound protein 2 and “Son of Sevenless” protein (Sos) and subsequent Ras/Raf/MEK/ERK signaling cascade activation (serine/threonine kinase/mitogen-activated protein kinase kinase/extracellular signal-regulated kinase). This important pathway is involved mainly in cell cycle progression and cell proliferation. 

Besides this complex pathway, EGFR is involved in other important cellular processes, such as regulating cytokines, hydrogen peroxide, or G protein-coupled receptors. In addition, NKA/Src can regulate the activity of phosphatidylinositol 3-kinase, which activates protein kinase B, leading to activation of several other proteins including the mammalian target of rapamycin, a kinase involved strongly in cell cycle progression. In summary, the binding of CGs to NKA stimulates diverse signaling pathways, many of which are directly involved in cell growth, proliferation, and cell death [38]. In addition to the main mechanism of NKA-binding, CGs can work through many other mechanisms. Both specific and unspecific modes of CGs action have been discovered so far, such as increasing the level of cholesterol by enhancing the activity of the key enzyme of cholesterol synthesis, 3-hydroxy-3-methylglutaryl-coenzyme A reductase [39], or altering the fluidity of the cell plasma membrane [40]. All this contributes to complex responses of cells to CG treatment, the outcomes of which might therefore be rather unpredictable. In addition to this complex mechanism of action of CGs, great variability of their biological actions comes also from NKA itself. Different isoforms of NKA subunits are expressed in different tissues [32] and CGs have different affinities for the particular isoforms. For example, digoxin and digitoxin are more selective to α2 and α3 than to α1 NKA isoform. On the contrary, ouabain is rather selective to α1 NKA isoform [41]. However, in some species this may vary. It has been described that in rats and mice, α1 isoform of NKA is resistant to ouabain [42]. The significant differences in CG action linked to α subunit NKA isoforms are caused also by the fact that the individual isoforms differ in their biological role. The α1 isoform exhibits both a pumping and signaling function, α2 plays a role in Ca^2+^ signaling and cardiac contractility, and α3 is linked to cardiac hypertrophy [43]. Recently, Singh et al. also reported a significant contribution of α subunit NKA isoforms in patients with bipolar disease, since changes in levels of particular subunits may alter levels of endogenous CGs and, thus, affect the disease development [44]. With knowledge of these facts, we must precisely consider the significance of the experimental data. The action of CGs must always be related to the levels of particular isoforms of the NKA α subunit and be always compared among individual species and tissues. 

## 3. Immunogenic Cell Death and CGs

Cell death is an extremely important process, which plays a role in homeostasis maintenance or tissue renewal. We distinguish accidental cell death, which occurs under extreme non-physiological conditions, and regulated cell death (RCD), which occurs most commonly both physiologically (also referred to as programmed cell death) and after exposure to exogenous stress stimuli. Physiologically, cell death plays an important role in human and animal development [46], tissue remodeling in plants [47], but occurs also in unicellular organisms, such as bacteria [48]. Stress-driven cell death ensures a right physiological equilibrium by eliminating irreversibly damaged cells. A lot of specific RCD molecular pathways (systematically described by the Nomenclature Committee on Cell Death, ref. [49]) have been described so far; however, morphologically, we distinguish three basic types: Type I, apoptosis, is typical by cytoplasmic shrinkage, chromatin condensation (pyknosis), nuclear fragmentation (karyorrhexis), and plasma membrane blebbing, all resulting in the creation of intact vesicles digested subsequently by macrophages. Type II, autophagy, is characterized by the formation of vesicles with fragments of cellular structures, which then fuse with lysosomes. Finally, type III RCD, necrosis, differs from the previous cell death types by practically direct shrinkage of the cytoplasmic membrane [49].

**Figure 3:**
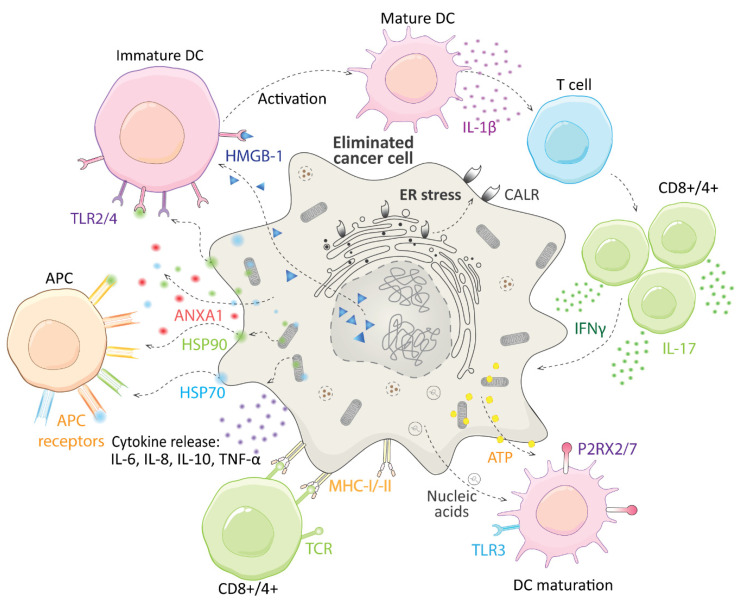
A schematic illustration of immunogenic cell death. The release of damage-associated molecular patterns (DAMPs), and activation of immune cells. ANXA1–annexin A1; APC–antigen-presenting cells; ATP–adenosine triphosphate; CALR–calreticulin; CD–a cluster of differentiation; DC –dendritic cells; ER–endoplasmic reticulum; HMGB-1–high mobility group box 1; HSP90/HSP70– heat shock protein 90/70; IFNγ–interferon γ; IL-interleukin; MHC I/-II–major histocompatibility complex I/II; TLR–Toll-like receptor; P2RX2/7-P2X purinoceptor 2/7; TNF α–tumor necrosis factor α.

Immunogenic cell death (ICD), in contrast to the aforementioned RCD types, leads to potent stimulation of immune and inflammatory response and even establishment of long-term immunological memory. Recently ICD has been extensively reviewed by Galluzzi et al. [50], thus, here we describe only basic molecular principles of ICD. ICD is a type of RCD, which leads to the complete immunological response of the organism to infected or malignant cells. Such cells express and present antigens derived from microorganisms (microbe-associated molecular patterns), or mutation processes in tumors (tumor neoantigens). These antigens are recognized by T cells. Healthy cells are usually not able to undergo ICD since their antigens are tolerated by the immune system. The most crucial molecules for the ICD process are damage-associated molecular patterns (DAMPs), which are usually constantly present in cells. They are first released, when exposed to certain stress factors, such as viruses, radiation, and photodynamic therapies, or some US Food and Drug Administration (FDA)-approved drugs (oxaliplatin [51], lurbinectedin [52], and cyclophosphamide [53]). Major DAMPs are ATP, calreticulin (CALR), high-mobility group box 1 (HMGB-1), type I interferon (IFN), annexin A1 (ANXA1), heat shock proteins 70 and 90 (HSP70, HSP90), or diverse nucleic acids, mainly cancer cell derived ones. DAMPs ensure mainly recruitment of antigen-presenting cells (APCs) and T cells, especially guiding the interaction between APCs and dying cells, promotion of maturation of APCs, and their capacity to affect cross-presentation (Figure 3). 

The release of the aforementioned DAMPs occurs for the most part after induction of endoplasmic reticulum (ER) stress by the above-discussed stimuli. It is now well established that ER stress is essential for CALR plasma membrane exposure, which is one of the most important steps in ICD induction. ER stress is a complex cellular pathway, which includes multiple proteins, such as protein kinase R-like ER kinase (PERK), which is phosphorylated at the beginning of the ER stress response and further phosphorylates eukaryotic translation initiation factor 2α (eIF2α). Phosphorylation of PERK is essential for cell death to be immunogenic because if PERK is depleted, the immunogenicity is lost. However, the process of CALR exposure is more complicated and includes multiple steps, extensively described in ref. [54]. Besides this direct pathway beginning with ER stress, an important role in ICD is played by reactive oxygen species (ROS), which, however, seem to be a double-edged sword in this process. On one hand, it has been proven that ROS-induced autophagy triggers ER stress [55], on the other hand, ROS oxidize HMBG-1 and weaken the function of T cells [56]. In general, ROS are needed for ICD induction, since antioxidants successfully impede ICD, but for the further flow of the ICD process, it is of benefit to remove them from the tumor microenvironment [54,56]. To sum up, for ICD induction, ER stress-causing, and ROS-generating compounds must be sought. These characteristics are possessed also by CGs, as we showed while discussing NKA signalosome. 

CGs were first identified to cause ICD by Menger et al. in 2012, who designed automated specific epifluorescence microscopy assays for ICD detection. They monitored the release and translocation of DAMPs, such as CALR, ATP, or HMGB-1, after exposure of human osteosarcoma cells (U-2 OS) to 1,040 FDA-approved and experimental drugs. The cells were stably producing CALR in a fusion with a green fluorescent protein (GFP), which enabled monitoring of CALR localization. ATP was stained with quinacrine and HMBG-1 release was detected by the decreased intensity of fluorescence emission of HMBG-1-GFP in the nucleus or increased fluorescence emission intensity in the extracellular area (detected by an enzyme-linked immunosorbent assay). In this screening, four CGs, digoxin, digitoxin, ouabain, and lanatoside A, were identified to be among the top ten most effective ICD inducers. The ability of CGs to cause CALR plasma membrane exposure and ATP and HMGB-1 release from cells to the extracellular area was further confirmed in a panel of human cancer cell lines, particularly cells from the cervix (HeLa), breast (MDA-MB 231), and colon adenocarcinoma (HCT 116), non–small-cell lung (A549), prostate (LNCaP), oral squamous (Cal27), and hepatocellular carcinoma (HepG2). In the same study, the authors confirmed that CGs induced ER stress, which was documented, for example, by phosphorylation of eIF2α. Moreover, it was shown that antioxidants inhibit the cytotoxicity of CGs, which means there is a strong correlation between their cytotoxicity and induction of ICD. Interestingly, Menger et al. confirmed the ability of cancer cells pretreated with CGs to vaccinate syngeneic mice against subsequent development of tumors consisting of the same type of cells, indicating that CGs can induce immunological memory [57]. 

Such “vaccination” property represents an extremely interesting tool in developing novel anticancer therapies. Most recently, Xiang et al. [58] confirmed this effect of CGs and implemented it in their combinatorial approach. They combined a standard cancer chemotherapeutic cisplatin bound to N-(2-hydroxypropyl) methacrylamide polymer (P-cis) with digoxin and investigated antitumor efficacy of such combination in B16F10 melanoma-bearing mice. They reported that P-cis was far more cytotoxic than cisplatin alone. Unfortunately, 5% of a tumor tissue remained viable after the treatment, thus, resulting in a subsequent recurrence of the tumor. However, in the group receiving P-cis in combination with digoxin, 100% cytotoxicity was confirmed, due to very potently induced ICD, which was demonstrated mainly by increased levels of cytotoxic CD8+ T lymphocytes. Besides, key components of ICD were identified in P-cis/digoxin-treated mice, namely CALR exposure on the plasma membrane and increase in extracellular ATP and HMGB-1. Considering that digoxin has very low systemic cytotoxicity, its property to induce ICD could be of extreme importance for future combinatorial anticancer applications with currently used drugs, which are not able to eliminate the tumor fully. 

## 4. Immunomodulatory Action of CGs in Cancer Treatment

The classic ICD pathway is, however, not the only mechanism, by which CGs impact the immune system and could, thus, be beneficial in cancer treatment. Da Silva et al. [59] studied the effects of ouabain on melanoma-bearing mice. They reported modulated levels of B and T cells, reduction of regulatory T cells, and anti-inflammatory properties of ouabain, for example, a reduction of cytokines such as interleukin 1β (IL-1β). Moreover, Shih et al. reported increased levels of T cells, macrophage phagocytosis, and natural killer cell levels after treatment of WEHI-3 (leukemia cells) tumor-bearing mice with ouabain [60]. The complex impact of ouabain on the immune system and inflammation has been reviewed in ref. [61]. The research group of Shih et al. also presented a study of a bufadienolide bufalin, which affected the immune system of mice comparably to ouabain [62]. Bufalin was also described to inhibit hepatocellular carcinoma (HCC) proliferation and migration. Yang et al. discovered a specific mechanism of bufalin immune system induction connected with reduced expression of the *APOBEC3F* gene (DNA dC->dU-editing enzyme). The authors bring evidence that the *APOBEC3F* gene is involved in the intestinal immune network of production of immunoglobulin A antibodies. As they found, certain members of this network, mainly polymeric immunoglobulin receptors or some chemokine receptors, are often upregulated in HCC and are linked with enhanced cell proliferation. By downregulating the *APOBEC3F* gene and decreasing cellular concentrations of the intestinal immune network receptors, bufalin is probably able to inhibit HCC growth [63].

Besides bufalin, other bufadienolides are also capable of immune system induction. Xie et al. extensively described immunogenic properties of cinobufagin, which is one of the active compounds in traditional Chinese medicine Chan Su prepared from the skin and parotid glands of Chinese toad, *Bufo bufo gargarizans*. Cinobufagin inhibits the maturation and production of cytokines and IL-1β, upregulates gene expression of human β-defensins, and induces secretion of human neutrophil peptides from neutrophils, which contributes mainly to its antimicrobial activity [64]. Cinobufagin is not the only bufadienolide from Chan Su with immunomodulatory properties. Cao et al. confirmed telocinobufagin to increase proliferation of natural killer cells and macrophages, enhance proliferation of splenocytes, and increase levels of IL-2, IL-12, interferon-γ, or tumor necrosis factor in vitro [65]. Telocinofufagin and bufalin were studied also by Yuan et al. together with two other bufadienolides, gamabufotalin, and arenobufagin. Interestingly, the latter two compounds exert a selective antiproliferative activity against cancer cell lines U-87 (glioblastoma) and SW1990 (pancreatic adenocarcinoma), since they were 3–5 times more toxic to these cell lines than to normal peripheral blood mononuclear cells. The other two tested compounds did not show such activity. Furthermore, gamabufotalin is capable of specifically downregulating the immunosuppressive regulatory T cells, which are often overexpressed in tumors, thus enhancing the antitumor immunity [66]. To sum up, CGs are potent immunostimulatory compounds, which with the use of potentiation of the immune system effectively inhibit cancer cell growth and tumor progression.

## 5. Immunomodulatory Action of CGs in Treatment of Autoimmune and Inflammatory Diseases

**Figure 4:**
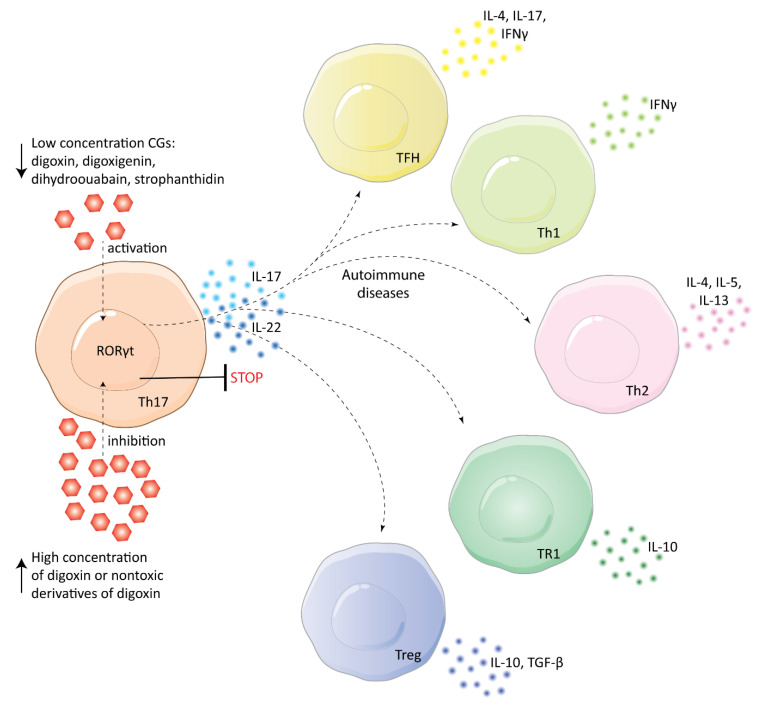
Cardiac glycosides at low nanomolar concentrations activate RORγt, whereas at high concentrations they cause RORγt transcriptional inhibition. This influences the production of interleukins (IL) 17 and 22 in T-helper cells expressing IL-17 (Th17). The interleukins promote autoimmune diseases by activating immune cells, such as follicular B-helper T cells (TFH), type 1 and type 2 T-helper cells (Th1, Th2), type 1 regulatory T cells (TR1), and regulatory T cells (Treg). These cells produce IL, interferon γ (IFNγ), or transforming growth factor-beta (TGF-β).

Besides cancer treatment, CGs could also be beneficial in the treatment of autoimmune diseases. The groundbreaking evidence in this field was published in 2011 by Huh et al., who studied the effects of digoxin on murine T-helper cells expressing IL-17 (Th17 cells). In their study, digoxin was reported to specifically inhibit the transcriptional activity of retinoic acid receptor-related orphan receptor γ thymus (RORγt), which is expressed only in Th17 cells and is essential for IL-17 transcription. Due to this inhibitory activity of digoxin, differentiation of Th17 cells was inhibited, while other types of T cells were not affected. Therefore, the onset of autoimmune reactions in mice was delayed and their severity was reduced. Importantly, Huh et al. also studied non-toxic derivatives of digoxin, 20,22-dihydrodigoxin-21,23-diol, and digoxin-21-salicylidene, which retained the affinity to RORγt and could thus be used as structural carriers for targeted therapy against autoimmune diseases [67]. Interestingly, Karaś et al. [68] reported the ability of cardenolides digoxigenin, dihydroouabain, and strophanthidin to activate RORγt in HepG2 cells, which is exactly the opposite activity to that reported by Huh et al. This important difference might be caused by structural differences among the compounds, but also by the concentrations of the CGs. Huh et al. experimented with rather high concentrations of digoxin (dozens of µM), thus, Karaś et al. [69] decided to study lower concentrations of the same compound, which would be also more suitable for clinical purposes. Surprisingly, they identified that digoxin activates RORγt in HepG2 cells similarly to the compounds in their previous study. Likewise, Karaś et al. reported induction of transcription of IL17A/F, IL21, IL22, IL23R, CCR4, and CCR6 genes in Th17 cells, which shows a very complex impact of digoxin on the cellular immune system (see Figure 4). 

Th17 cells play an important role in inflammation and development of different inflammation-related diseases. Untreatable abdominal aortic aneurysm (AAA) occurring commonly in elderly males is one of them. This disease is characterized by local enlargements in the infrarenal aorta, the rupture of which causes death in 75 to 95% of patients. The rupture of the aorta in AAA is directly linked to inflammation, which is caused mainly by Th17 cells. Thus, Wei et al. [70] proposed digoxin to be a potential treatment for AAA. Their experiments on the apolipoprotein E-deficient mice model confirmed digoxin efficacy since high doses of digoxin (40 μg per day per mouse) decreased AAA incidence twice in comparison to the control model. However, digoxin was not able to completely prevent AAA development and its concentration needed for RORγt inhibition was much higher (>10 μM) than the half-maximal cytotoxic concentration (dozens to hundreds of nM for various non-malignant cell lines after 48 h) [71]. Therefore, nontoxic derivatives of digoxin are needed to achieve a better therapeutic outcome, as well as to further study the relationships between Th17 and AAA. In general, IL-17 and Th17 are the basis for the very large clinical potential of digoxin. Due to RORγt inhibition and subsequent suppression of Th17 cells, digoxin could be beneficial also for the treatment of rheumatoid arthritis, atherosclerosis, or colitis [72,73,74]. 

When talking about digoxin and inflammation, the effect of CGs on sterile (pathogen-free) inflammation in the liver, particularly non-alcoholic steatohepatitis (NASH), must be mentioned. Ouyang et al. [75] reported their pivotal research on this topic in 2018 and only three years after, clinical trials of digoxin in the treatment of sterile inflammation (recruiting) [76] and NASH (not yet recruiting) [77] are in progress. Thus, these conditions joined the clinically tested portfolio of digoxin, which was so far limited to heart conditions and cancer. In the study of Ouyang et al., the action of digoxin on NASH was reported to be based on suppressing oxidative stress and maintaining cellular redox homeostasis. This was ensured by suppressing hypoxia-induced factor 1 α via binding to pyruvate kinase M2 (PKM2). Furthermore, digoxin was able to inhibit transcription of PKM2-dependent genes by impeding the binding of PKM2 to histones. By binding to PKM2, digoxin ensured also downregulation of transcription of nuclear factor kappa-light-chain-enhancer of activated B cells (NFκB) and, most importantly, lowered the formation of cytotoxic ROS in a cell. Therefore, the ratio of dead cells was lower and the progression of sterile inflammation was minimal as showed on mice models. Given this pivotal evidence, digoxin represents a potential treatment for NASH or sterile inflammation, and further studies on human patients are recommended to confirm this hypothesis. 

However, not only digoxin proceeded into clinical trials in connection with inflammation. Related cardenolide digitoxin was tested on patients with cystic fibrosis (CF) as an agent potentially able to reduce airway inflammation. The major inflammatory marker present in sputum and fluid of CF patients is IL-8, the transcription of which is mediated by NFκB. Digitoxin, similarly to digoxin, was reported to suppress the activity of NFκB. The report from the preliminary clinical study of digitoxin (0.1 mg daily for 28 days) on CF patients showed good tolerability of this drug and a slight decrease of neutrophil levels; however, it did not show a significant decrease of inflammatory markers, mainly IL-8, in sputum. Moreover, it took a long time (up to 4 weeks) to reach a steady-state of therapeutic concentration of digitoxin in serum. However, the study of nasal immune response mRNA levels indicated significant transcriptional effects of digitoxin in patients. Mainly affected were levels of IL-8 and IL-6 mRNAs, which corresponds to the previous knowledge. Thus, it seems that digitoxin could be effective against airway inflammation, but observation longer than 28 days is needed for confirmation [78]. Besides digitoxin, bufalin was also reported as a potential airway inflammation drug. Zhakeer et al. studied its effects on asthmatic ovalbumin sensitized BALB/c mice and observed a significant decrease in levels of immune-inflammatory cells, such as macrophages, eosinophils, lymphocytes, or neutrophils, in bronchoalveolar lavage fluid. Additionally, levels of IL-4, IL-5, and IL-13 in serum were lower. The main reason for such activity was confirmed again as inhibition of NFκB [79]. Very similar outcomes were observed by Galvão et al. for ouabain [80]. 

In addition to the aforementioned inflammatory conditions, neuroinflammation could be also potentially treatable with CGs. Jansson et al. [81] developed a screening system for the identification of the compounds targeting inflammation in blood–brain barrier-derived pericytes and endothelia. These cells are crucial for leucocyte extravasation during neurodegenerative diseases. By screening 1280 FDA-approved drugs, the authors identified digoxin and lanatoside C as inflammation modifiers in the aforementioned cells. The two CGs potently inhibited IL-1β-induced secretion of cytokines and chemokines, such as (C-C motif) ligand 2, intercellular adhesion molecule-1, soluble vascular cell adhesion molecule-1, or fractalkine. All inflammatory secretions were abolished in endothelia, but in pericytes, increased levels of IL-6 were observed. The differences might be caused by cell-type-specific expression of NKA (as discussed in Section 2). To further confirm the anti-neuroinflammatory efficacy of CGs, Jansson et al. studied their effects on an ex vivo multicellular culture model explanted from post-mortem brains. This model of leptomeningeal and choroidal plexus tissue was shown to be similarly susceptible to digoxin and lanatoside C as pericytes and endothelia in vitro. Importantly, none of the CGs inhibited the inflammatory response fully, the basic level of inflammatory components needed for homeostasis was preserved. The mechanism of action of digoxin and lanatoside C must yet be elucidated; however, the authors propose a certain role of NFκB or CCAAT/enhancer-binding protein δ, which downregulates IL-1β and is present in pericytes. Neuroinflammation is a factor that highly increases the progression of neurodegenerative diseases. If the efficacy of CGs against this condition was proven, it would be a breakthrough in the treatment of many such diseases, since so far there are only a few effective drugs in the field and all the in vitro confirmed anti-neurodegenerative agents failed in in vivo preclinical and clinical testing. A similar premise holds good for other aforementioned conditions (Table 1). CGs, thus, represent extremely promising drug candidates and it is very worthy to study them further. 

## 6. Conclusions

CGs are potent NKA inhibitors, which have long been used in cardiac medicine. However, due to the involvement of NKA in the very complex cellular signaling, CGs have recently attracted increased attention as potential therapeutics for various other indications. Currently, they are being clinically tested mostly against cancer, since by triggering the NKA signalosome, CGs can induce apoptosis. However, what makes CGs exceptional among other anticancer drug candidates such as antimitotics [82,83]? In this review article, we showed that the great advantage of CGs in anticancer treatment is the induction of ICD. Owing to this characteristic, CGs might be an excellent option in combinatorial treatment, as they can efficiently eliminate cancer cells surviving after conventional treatment. Furthermore, they might be useful in anticancer vaccination, as they have been shown to induce immunological memory. We also provided an overview of other potential therapeutic applications of CGs, which are linked with immunomodulatory properties, namely treating inflammation and autoimmune diseases. CGs can inhibit Th17 activity and IL-17 release through inhibition of the transcriptional activity of RORγt, by which they significantly reduce the inflammatory response. This, together with suppressing NFκB activity, makes CGs potentially suitable for the treatment of sterile inflammation, NASH, or airway inflammation complicating cystic fibrosis. In addition, due to IL-1β inhibition, CGs could also be beneficial in neuroinflammation and, thus, they represent a hope for the treatment of neurodegenerative diseases. To sum up, CGs could be potentially implemented in a broad portfolio of therapies, and, therefore, it is of great importance to further study these compounds, elucidate their mechanism of immunomodulatory action, and move them further into clinical trials.

## Figures and Tables

**Table 1:** Potential therapeutic opportunities for cardiac glycosides (CGs) and the main mechanism of action against particular conditions.

CG	Condition	Mechanism of Action	Reference
Digoxin, digitoxin, ouabain, bufalin gamabufotalin arenobufagin	Cancer	Induction of ICD	[57,60,63,66]
Cinobufagin	Microbial infection	Upregulating IL-1β	[64]
Digoxin	Abdominal aortic aneurysm	RORγt inhibition, suppressing Th17 differentiation	[70]
	Rheumatoid arthritis		[73]
	Colitis		[72]
	Atherosclerosis		[70]
	NASH	Suppressing hypoxia-induced factor 1 α, NFκB transcription, and ROS through binding to PKM2	[74]
Digitoxin	Cystic fibrosis	Suppressing NFκB, downregulating IL-8	[78]
Bufalin, ouabain	Airway inflammation (asthma)	Suppressing NFκB	[79,80]

ICD-immunogenic cell death; IL-1β-interleukin 1β; RORγt-retinoic acid receptor-related orphan receptor γ thymus; NASH-non-alcoholic steatohepatitis; NFκB-nuclear factor κB; ROS-reactive oxygen species; PKM2-protein kinase M2; IL-8-interleukin 8.

## Data Availability

Not applicable.

## Abbreviations

A549	Human cells from non–small-cell lung carcinoma
AAA	Abdominal aortic aneurysm
ANXA1	Annexin A1
AP1	Transcription factor
APC	Antigen-presenting cells
APOBEC3F DNA	dC->dU-editing enzyme
ATP	Adenosine triphosphate
B16F10	Mouse cells from melanoma
Cal27	Human cells from oral squamous carcinoma
CALR	Calreticulin
CD	Cluster of differentiation
CF	Cystic fibrosis
CG	Cardiac glycosides
DAMP	Damage-associated molecular patterns
DC	Dendritic cell
EGFR	Epidermal growth factor receptor
ER	Endoplasmic reticulum
ERK	Extracellular signal-regulated kinase
FDA	US Food and Drug Administration
GFP	Green fluorescent protein
GRB2	Growth factor receptor-bound protein 2
HCC	Human cells from hepatocellular carcinoma
HCT 116	Human cells colon adenocarcinoma
HeLa	Human cells from cervical carcinoma
HepG2	Human cells from hepatocellular carcinoma
HMGB-1	High-mobility group box 1
HSP70: HSP90	Heat shock proteins 70 and 90
ICD	Immunogenic cell death
IFNγ	Interferon γ
IL	Interleukin
IP_3_	Inositol triphosphate
IP3R	Inositol triphosphate receptor
LNCaP	Human cells from prostate carcinoma
MDA-MB 231	Human cells from breast carcinoma
MEK	Mitogen-activated protein kinase kinase
MHC-I/-II	Major histocompatibility complex I/II
NASH	Non-alcoholic steatohepatitis
NF-κB	Nuclear factor kappa-light-chain-enhancer of activated B cells
NKA	Na^+^/K^+^-ATPase
P2RX2/7	P2X purinoceptor 2/7
PCD	Programmed cell death
PERK	Protein kinase R-like ER kinase
PKC	Protein kinase C
PKM2	Pyruvate kinase M2
PLC	Phospholipase C
Raf	Serine/threonine kinase
Ras	Rat sarcoma protein
Rcd	Regulated cell death
RORγt	Receptor-related orphan receptor γ thymus
ROS	Reactive oxygen species
SHC	Src homology 2 domain-containing transforming protein 1
SOS	Son of Sevenless
Src	Non-receptor tyrosine kinase
TLR	Toll-like receptor
TNF α	Tumor necrosis factor α
U-2 OS	Human cells derived from osteosarcoma
WEHI-3	Human leukemia cells

## References

[1] Sung H., Ferlay J., Siegel R.L., Laversanne M., Soerjomataram I., Jemal A., Bray F. (2021). Global cancer statistics 2020: GLOBOCAN estimates of incidence and mortality worldwide for 36 cancers in 185 countries. CA Cancer J. Clin..

[2] Bejcek J., Spiwok V., Kmonickova E., Ruml T., Rimpelova S. (2021). Cardiac glycosides: On their therapeutic potential for cancer treatment. Chem. Listy.

[3] Garcia I.J.P., de Oliveira G.C., Valadares J.M.D., Banfi F.F., Andrade S.N., Freitas T.R., Filho E.D.S.M., Santos H.D.L., Vieira G.M., Chaves M.H. (2019). New bufadienolides extracted from *Rhinella Marina* inhibit Na,K-ATPase and induce apoptosis by activating caspases 3 and 9 in human breast and ovarian cancer cells. Steroids.

[4] Geng X., Wang F., Tian D., Huang L., Streator E., Zhu J., Kurihara H., He R., Yao X., Zhang Y. (2020). Cardiac glycosides inhibit cancer through Na/K-ATPase-dependent cell death induction. Biochem. Pharmacol..

[5] Kanwal N., Rasul A., Hussain G., Anwar H., Shah M.A., Sarfraz I., Riaz A., Batool R., Shahbaz M., Hussain A. (2020). Oleandrin: A bioactive phytochemical and potential cancer killer via multiple cellular signaling pathways. Food Chem. Toxicol..

[6] Lan Y.L., Zou Y.J., Lou J.C., Xing J.S., Wang X., Zou S., Ma B.B., Ding Y., Zhang B. (2019). The sodium pump α1 subunit regulates bufalin sensitivity of human glioblastoma cells through the p53 signaling pathway. Cell Biol. Toxicol..

[7] Leu W.J., Wang C.T., Hsu J.L., Chen I.S., Chang H.S., Guh J.H. (2020). Ascleposide, a natural cardenolide, induces anticancer signaling in human castration-resistant prostatic cancer through Na^+^/K^+^-ATPase internalization and tubulin acetylation. Prostate.

[8] Michalak K., Rárová L., Kubala M., Štenclová T., Strnad M., Wicha J. (2020). Synthesis and evaluation of Na^+^/K^+^-ATP-ase inhibiting and cytotoxic in vitro activities of oleandrigenin and its selected 17 beta-(butenolidyl)-and 17 beta-(3-furyl)- analogues. Eur. J. Med. Chem..

[9] Reddy D., Ghosh P., Kumavath R. (2020). Strophanthidin attenuates MAPK, PI3K/AKT/mTOR, and Wnt/beta-catenin signaling pathways in human cancers. Front. Oncol..

[10] Reddy D., Kumavath R., Barh D., Azevedo V., Ghosh P. (2020). Anticancer and antiviral properties of cardiac glycosides: A review to explore the mechanism of actions. Molecules.

[11] Reddy D., Kumavath R., Tan T.Z., Ampasala D.R., Kumar A.P. (2020). Peruvoside targets apoptosis and autophagy through MAPK Wnt/beta-catenin and PI3K/AKT/mTOR signaling pathways in human cancers. Life Sci..

[12] Ren Y., Ribas H.T., Heath K., Wu S., Ren J., Shriwas P., Chen X., Johnson M.E., Cheng X., Burdette J.E. (2020). Na^+^/K^+^-ATPase-targeted cytotoxicity of (+)-digoxin and several semisynthetic derivatives. J. Nat. Prod..

[13] Rimpelová S., Zimmermann T., Drašar P.B., Dolenský B., Bejček J., Kmoníčková E., Cihlářová P., Gurská S., Kuklíková L., Hajdůch M. (2021). Steroid glycosides hyrcanoside and deglucohyrcanoside: On isolation, structural identification, and anticancer activity. Foods.

[14] Schneider N.F.Z., Menegaz D., Dagostin A.L.A., Persich L., Rocha S.C., Ramos A.C.P., Cortes V.F., Fontes C.F.L., de Pádua R.M., Munkert J. (2021). Cytotoxicity of glucoevatromonoside alone and in combination with chemotherapy drugs and their effects on Na^+^,K^+^-ATPase and ion channels on lung cancer cells. Mol. Cell. Biochem..

[15] Song Y., Lee S.Y., Kim S., Choi I., Kim S.H., Shum D., Heo J., Kim A.R., Kim K.M., Seo H.R. (2020). Inhibitors of Na^+^/K^+^ ATPase exhibit antitumor effects on multicellular tumor spheroids of hepatocellular carcinoma. Sci. Rep..

[16] Sun X., Ng T.T.H., Sham K.W.Y., Zhang L., Chan M.T.V., Wu W.K.K., Cheng C.H.K. (2019). Bufalin, a traditional chinese medicine compound, prevents tumor formation in two murine models of colorectal cancer. Cancer Prev. Res..

[17] Wang F., Liu L., Tong Y., Li L., Liu Y., Gao W.Q. (2020). Proscillaridin A slows the prostate cancer progression through triggering the activation of endoplasmic reticulum stress. Cell Cycle.

[18] Wang Y., Ma Q., Zhang S., Liu H., Zhao B., Du B., Wang W., Lin P., Zhang Z., Zhong Y. (2020). Digoxin enhances the anticancer effect on non-small cell lung cancer while reducing the cardiotoxicity of adriamycin. Front. Pharmacol..

[19] Bhusare B.P., John C.K., Bhatt V.P., Nikam T.D. (2018). In vitro propagation of *Digitalis lanata* Ehrh. through direct shoot regeneration—A source of cardiotonic glycosides. Ind. Crop Prod..

[20] Curfman G. (2020). Digitalis glycosides for heart rate control in atrial fibrillation. JAMA.

[21] Fujino T., Kuroda M., Matsuo Y., Kubo S., Tamura C., Sakamoto N., Mimaki Y., Hayakawa M. (2015). Cardenolide glycosides from the seeds of Digitalis purpurea exhibit carcinoma-specific cytotoxicity toward renal adenocarcinoma and hepatocellular carcinoma cells. Biosci. Biotechnol. Biochem..

[22] Kirmizibekmez H., Masullo M., Festa M., Capasso A., Piacente S. (2014). Steroidal glycosides with antiproliferative activities from *Digitalis trojana*. Phytother. Res..

[23] Dunn D.E., He D.N., Yang P., Johansen M., Newman R.A., Lo D.C. (2011). In vitro and in vivo neuroprotective activity of the cardiac glycoside oleandrin from *Nerium oleander* in brain slice-based stroke models. J. Neurochem..

[24] Balderas-López J.L., Barbonetti S., Pineda-Rosas E.L., Tavares-Carvalho J.C., Navarrete A. (2019). Cardiac glycosides from *Cascabela thevetioides* by HPLC-MS analysis. Rev. Bras. Farmacogn..

[25] Kohls S., Scholz-Böttcher B.M., Teske J., Zark P., Rullkötter J. (2012). Cardiac glycosides from yellow oleander (*Thevetia peruviana*) seeds. Phytochemistry.

[26] Siddiqui B.S., Khatoon N., Begum S., Farooq A.D., Qamar K., Bhatti H.A., Ali S.K. (2012). Flavonoid and cardenolide glycosides and a pentacyclic triterpene from the leaves of Nerium oleander and evaluation of cytotoxicity. Phytochemistry.

[27] Mohammadi S., French S.S., Neuman-Lee L.A., Durham S.L., Kojima Y., Mori A., Brodie E.D., Savitzky A.H. (2017). Corticosteroid responses of snakes to toxins from toads (bufadienolides) and plants (cardenolides) reflect differences in dietary specializations. Gen. Comp. Endocrinol..

[28] Qi J., Zulfiker A.H.M., Li C., Good D., Wei M.Q. (2018). The Development of toad toxins as potential therapeutic agents. Toxins.

[29] Buckalew V.M. (2015). Endogenous digitalis-like factors: An overview of the history. Front. Endocrinol..

[30] Buckalew V.M. (2018). Role of endogenous digitalis-like factors in the clinical manifestations of severe preeclampsia: A systematic review. Clin. Sci..

[31] Melero C.P., Medarde M., San Feliciano A. (2000). A short review on cardiotonic steroids and their aminoguanidine analogues. Molecules.

[32] Bejček J., Spiwok V., Kmoníčková E., Rimpelová S. (2021). Na^+^/K^+^-ATPase revisited: On its mechanism of action, role in cancer, and activity modulation. Molecules.

[33] Smith T.W. (1988). Digitalis. Mechanisms of action and clinical use. N. Engl. J. Med..

[34] Kepp O., Menger L., Vacchelli E., Adjemian S., Martins I., Ma Y., Sukkurwala A.Q., Michaud M., Galluzzi L., Zitvogel L. (2012). Anticancer activity of cardiac glycosides. Oncoimmunology.

[35] Paula S., Tabet M.R., Ball W.J. (2005). Interactions between cardiac glycosides and sodium/potassium-ATPase: Three-dimensional structure-activity relationship models for ligand binding to the E2-Pi form of the enzyme versus activity inhibition. Biochemistry..

[36] Lopina O.D., Tverskoi A.M., Klimanova E.A., Sidorenko S.V., Orlov S.N. (2020). Ouabain-induced cell death and survival. Role of α1-Na,K-ATPase-mediated signaling and [Na^+^]_i_/[K^+^]_i_-dependent gene expression. Front. Phys..

[37] Pratt R.D., Brickman C.R., Cottrill C.L., Shapiro J.I., Liu J. (2018). The Na/K-ATPase Signaling: From specific ligands to general reactive oxygen species. Int. J. Mol. Sci..

[38] Cui X., Xie Z. (2017). Protein interaction and Na/K-ATPase-mediated signal transduction. Molecules.

[39] Campia I., Gazzano E., Pescarmona G., Ghigo D., Bosia A., Riganti C. (2009). Digoxin and ouabain increase the synthesis of cholesterol in human liver cells. Cell. Mol. Life Sci..

[40] Manna S.K., Sreenivasan Y., Sarkar A. (2006). Cardiac glycoside inhibits IL-8-induced biological responses by downregulating IL-8 receptors through altering membrane fluidity. J. Cell. Physiol..

[41] Katz A., Lifshitz Y., Bab-Dinitz E., Kapri-Pardes E., Goldshleger R., Tal D.M., Karlish S.J. (2010). Selectivity of digitalis glycosides for isoforms of human Na,K-ATPase. J. Biol. Chem..

[42] Wansapura A.N., Lasko V., Xie Z., Fedorova O.V., Bagrov A.Y., Lingrel J.B., Lorenz J.N. (2009). Marinobufagenin enhances cardiac contractility in mice with ouabain-sensitive alpha1 Na^+^-K^+^-ATPase. Am. J. Physiol. Heart Circ. Physiol..

[43] Liu L., Wu J., Kennedy D.J. (2016). Regulation of cardiac remodeling by cardiac Na^+^/K^+^-ATPase isoforms. Front. Physiol..

[44] Singh S.V., Fedorova O.V., Wei W., Rosen H., Horesh N., Ilani A., Lichtstein D. (2020). Na^+^, K^+^-ATPase α isoforms and endogenous cardiac steroids in prefrontal cortex of bipolar patients and controls. Int. J. Mol. Sci..

[45] Xie Z., Cai T. (2003). Na^+^-K^+^--ATPase-mediated signal transduction: From protein interaction to cellular function. Mol. Interv..

[46] Kutscher L.M., Shaham S. (2017). Non-apoptotic cell death in animal development. Cell Death Differ..

[47] Gunawardena A.H.L.A.N. (2008). Programmed cell death and tissue remodelling in plants. J. Exp. Bot..

[48] Allocati N., Masulli M., Di Ilio C., De Laurenzi V. (2015). Die for the community: An overview of programmed cell death in bacteria. Cell Death Dis..

[49] Galluzzi L., Vitale I., Aaronson S.A., Abrams J.M., Adam D., Agostinis P., Alnemri E.S., Altucci L., Amelio I., Andrews D.W. (2018). Molecular mechanisms of cell death: Recommendations of the Nomenclature Committee on Cell Death 2018. Cell Death Differ..

[50] Galluzzi L., Vitale I., Warren S., Adjemian S., Agostinis P., Buque Martinez A., Chan T.A., Coukos G., Demaria S., Deutsch E. (2020). Consensus guidelines for the definition, detection and interpretation of immunogenic cell death. J. Immunother. Cancer.

[51] Sun F., Cui L., Li T., Chen S., Song J., Li D. (2019). Oxaliplatin induces immunogenic cells death and enhances therapeutic efficacy of checkpoint inhibitor in a model of murine lung carcinoma. J. Recept. Signal. Transduct. Res..

[52] Kepp O., Zitvogel L., Kroemer G. (2020). Lurbinectedin: An FDA-approved inducer of immunogenic cell death for the treatment of small-cell lung cancer. Oncoimmunology.

[53] Du B., Waxman D.J. (2020). Medium dose intermittent cyclophosphamide induces immunogenic cell death and cancer cell autonomous type I interferon production in glioma models. Cancer Lett..

[54] Panaretakis T., Kepp O., Brockmeier U., Tesniere A., Bjorklund A.C., Chapman D.C., Durchschlag M., Joza N., Pierron G., van Endert P. (2009). Mechanisms of pre-apoptotic calreticulin exposure in immunogenic cell death. EMBO J..

[55] Garg A.D., Dudek A.M., Ferreira G.B., Verfaillie T., Vandenabeele P., Krysko D.V., Mathieu C., Agostinis P. (2013). ROS-induced autophagy in cancer cells assists in evasion from determinants of immunogenic cell death. Autophagy.

[56] Deng H., Yang W., Zhou Z., Tian R., Lin L., Ma Y., Song J., Chen X. (2020). Targeted scavenging of extracellular ROS relieves suppressive immunogenic cell death. Nat. Commun..

[57] Menger L., Vacchelli E., Adjemian S., Martins I., Ma Y., Shen S., Yamazaki T., Sukkurwala A.Q., Michaud M., Mignot G. (2012). Cardiac glycosides exert anticancer effects by inducing immunogenic cell death. Sci. Transl. Med..

[58] Xiang Y., Chen L., Li L., Huang Y. (2020). Restoration and enhancement of immunogenic cell death of cisplatin by coadministration with digoxin and conjugation to HPMA copolymer. ACS Appl. Mater. Interfaces.

[59] da Silva J.M.C., Campos M.L.A., Teixeira M.P., Faustino R.D.S., Aleixo R.C., Cavalcante F.J.P., Gomes L.R.O., de Albuquerque L.Z., Azevedo A.D.N., Cabral V.R. (2020). Ouabain pre-treatment modulates B and T lymphocytes and improves survival of melanoma-bearing animals. Int. Immunopharmacol..

[60] Shih Y.L., Shang H.S., Chen Y.L., Hsueh S.C., Chou H.M., Lu H.F., Lee M.Z., Hou H.T., Chuang Y.Y., Lee M.H. (2019). Ouabain promotes immune responses in WEHI-3 cells to generate leukemia mice through enhancing phagocytosis and natural killer cell activities *in vivo*. Environ. Toxicol..

[61] Cavalcante-Silva L.H.A., Lima E.D., Carvalho D.C.M., de Sales-Neto J.M., Alves A.K.D.A., Galvao J.G.F.M., da Silva J.S.D.F., Rodrigues-Mascarenhas S. (2017). Much more than a cardiotonic steroid: Modulation of inflammation by ouabain. Front. Physiol..

[62] Shih Y.L., Chou J.S., Chen Y.L., Hsueh S.C., Chung H.Y., Lee M.H., Chen C.P., Lee M.Z., Hou H.T., Lu H.F. (2018). Bufalin enhances immune responses in leukemic mice through enhancing phagocytosis of macrophage in vivo. In Vivo.

[63] Yang Z., Tao Y., Xu X., Cai F., Yu Y., Ma L. (2018). Bufalin inhibits cell proliferation and migration of hepatocellular carcinoma cells via APOBEC3F induced intestinal immune network for IgA production signaling pathway. Biochem. Biophys. Res. Commun..

[64] Xie S., Spelmink L., Codemo M., Subramanian K., Pütsep K., Henriques-Normark B., Olliver M. (2016). Cinobufagin modulates human innate immune responses and triggers antibacterial activity. PLoS ONE.

[65] Cao Y., Song Y., An N., Zeng S., Wang D., Yu L., Zhu T., Zhang T., Cui J., Zhou C. (2009). The effects of telocinobufagin isolated from Chan Su on the activation and cytokine secretion of immunocytes in vitro. Fundam. Clin. Pharmacol..

[66] Yuan B., He J., Kisoh K., Hayashi H., Tanaka S., Si N., Zhao H.Y., Hirano T., Bian B., Takagi N. (2016). Effects of active bufadienolide compounds on human cancer cells and CD4+CD25+Foxp3+ regulatory T cells in mitogen-activated human peripheral blood mononuclear cells. Oncol. Rep..

[67] Huh J.R., Leung M.W.L., Huang P., Ryan D.A., Krout M.R., Malapaka R.R.V., Chow J., Manel N., Ciofani M., Kim S.V. (2011). Digoxin and its derivatives suppress TH17 cell differentiation by antagonizing RORγt activity. Nature.

[68] Karaś K., Sałkowska A., Walczak-Drzewiecka A., Ryba K., Dastych J., Bachorz R.A., Ratajewski M. (2018). The cardenolides strophanthidin, digoxigenin and dihydroouabain act as activators of the human ROR gamma/ROR gamma T receptors. Toxicol. Lett..

[69] Karaś K., Sałkowska A., Sobalska-Kwapis M., Walczak-Drzewiecka A., Strapagiel D., Dastych J., Bachorz R.A., Ratajewski M. (2018). Digoxin, an overlooked agonist of RORγ/RORγT. Front. Pharmacol..

[70] Wei Z., Wang Y., Zhang K., Liao Y., Ye P., Wu J., Wang Y., Li F., Yao Y., Zhou Y. (2014). Inhibiting the Th17/IL-17A-related inflammatory responses with digoxin confers protection against experimental abdominal aortic aneurysm. Arterioscler. Thromb. Vasc. Biol..

[71] Calderón-Montaño J.M., Burgos-Morón E., López-Lázaro M. (2014). The in vivo antitumor activity of cardiac glycosides in mice xenografted with human cancer cells is probably an experimental artifact. Oncogene.

[72] Tani S., Takano R., Tamura S., Oishi S., Iwaizumi M., Hamaya Y., Takagaki K., Nagata T., Seto S., Horii T. (2017). Digoxin attenuates murine experimental colitis by downregulating Th17-related cytokines. Inflamm. Bowel. Dis..

[73] Saeed H., Mateen S., Moin S., Khan A.Q., Owais M. (2020). Cardiac glycoside digoxin ameliorates pro-inflammatory cytokines in PBMCs of rheumatoid arthritis patients in vitro. Int. Immunopharmacol..

[74] Shi H., Mao X., Zhong Y., Liu Y., Zhao X., Yu K., Zhu R., Wei Y., Zhu J., Sun H. (2016). Digoxin reduces atherosclerosis in apolipoprotein E-deficient mice. Br. J. Pharmacol..

[75] Ouyang X., Han S.N., Zhang J.Y., Evangelos D., Nemeth B.T., Pacher P., Feng D., Bataller R., Cabezas J., Stärkel P. (2018). Digoxin suppresses pyruvate kinase M2-promoted HIF-1α transactivation in steatohepatitis. Cell Metab..

[76] ClinicalTrials.gov. https://www.clinicaltrials.gov/ct2/show/NCT03559868?term=digoxin&recrs=abdf&draw=2&rank=7.

[77] ClinicalTrials.gov. https://www.clinicaltrials.gov/ct2/show/NCT04216693?term=digoxin&recrs=abdf&draw=2&rank=6.

[78] Zeitlin P.L., Diener-West M., Callahan K.A., Lee S., Talbot C.C., Pollard B., Boyle M.P., Lechtzin N. (2017). Digitoxin for airway inflammation in cystic fibrosis: Preliminary assessment of safety, pharmacokinetics, and dose Finding. Ann. Am. Thorac. Soc..

[79] Zhakeer Z., Hadeer M., Tuerxun Z., Tuerxun K. (2017). Bufalin inhibits the inflammatory effects in asthmatic mice through the suppression of nuclear factor-kappa B activity. Pharmacology.

[80] Galvão J.G.F.M., Cavalcante-Silva L.H.A., Carvalho D.C.M., Ferreira L.K.D.P., Monteiro T.M., Alves A.F., Ferreira L.A.M.P., Gadelha F.A.A.F., Piuvezam M.R., Rodrigues-Mascarenhas S. (2017). Ouabain attenuates ovalbumin-induced airway inflammation. Inflamm. Res..

[81] Jansson D., Dieriks V.B., Rustenhoven J., Smyth L.C.D., Scotter E., Aalderink M., Feng S., Johnson R., Schweder P., Mee E. (2021). Cardiac glycosides target barrier inflammation of the vasculature, meninges and choroid plexus. Commun. Biol..

[82] Škubník J., Jurášek M., Ruml T., Rimpelová S. (2020). Mitotic poisons in research and medicine. Molecules.

[83] Škubník J., Pavlíčková V., Ruml T., Rimpelová S. (2021). Current perspectives on taxanes: Focus on their bioactivity, delivery and combination therapy. Plants (Basel).

